# Integrated Behavioral Profiles of Physical Activity and Dietary Intake in Young Adults and Their Associations with Lower Limb Injury Occurrence

**DOI:** 10.3390/nu17203196

**Published:** 2025-10-11

**Authors:** Jarosław Domaradzki

**Affiliations:** Department of Biological Principles of Physical Activity, Wroclaw University of Health and Sport Sciences, 51-612 Wrocław, Poland; jaroslaw.domaradzki@awf.wroc.pl

**Keywords:** behavioral clustering, lifestyle factors, energy balance, exercise-related injuries, university students

## Abstract

**Background/Objectives**: To delineate integrated lifestyle profiles combining physical activity (PA) and dietary intake (DI) and test their links with lower limb injury in physically active young adults. **Methods**: We analyzed a cross-sectional convenience sample of university students (men: *n* = 91, 20.5 ± 1.0 years; women: *n* = 118, 20.3 ± 0.8 years). PA (IPAQ) and DI (QEB) were assessed alongside self-reported injuries. Latent class modeling derived PA–DI profiles. Injury prevalence across profiles was compared (χ^2^), and logistic regression examined injury odds adjusting for sex, age, and BMI. **Results**: Four profiles emerged. Two reflected less healthy patterns (Profiles 2–3) and two healthier ones (Profiles 1, 4). Profile 4 showed higher vegetables/legumes/fermented milk and lower fast food/sugary drinks; Profile 3 combined greater sitting and fried/sweetened items with lower walking/milk intake. Overall injury prevalence was 56.9%, ranging from 44.1% (Profile 2) to 66.7% (Profile 4 exceeded Profile 2 in pairwise comparison (χ^2^ (1) = 5.08, *p* = 0.024)). In adjusted models, men had higher injury odds (OR = 1.94, 95% CI: 1.09–3.48, *p* = 0.025); profile membership was not independently predictive, and profile × sex interactions were null. **Conclusions**: Young adults cluster into distinct PA–DI patterns that differ behaviorally, but sex—rather than profile—was the most consistent correlate of injury. Prevention should integrate lifestyle screening with sex-specific strategies.

## 1. Introduction

Young adulthood is a critical life stage for establishing physical activity (PA) patterns that influence long-term health [1]. At the same time, athletes in this period are particularly prone to injury due to hormonal, musculoskeletal, and neurocognitive changes that create intrinsic risk factors [2]. Musculoskeletal injuries are common in physically active populations, with about one-quarter reporting incidents—most of them activity-related and affecting the lower extremities [3,4]. Beyond physical harm, injuries often undermine return-to-play confidence, increase reinjury risk, and contribute to psychological distress [5]. Severe cases may even lead one-third of individuals to permanently abandon exercise [3].

Diet and PA are both key determinants of health: diet supports musculoskeletal recovery, while PA enhances cardiovascular and mental well-being [6]. Yet, interventions targeting them separately have produced limited effects against obesity and metabolic disease [7]. Evidence suggests synergistic benefits when they are combined; for instance, low adherence to the Mediterranean diet together with low PA increases mortality risk more than either factor alone [8]. Although our study did not assess adherence to the Mediterranean diet, this example illustrates the importance of examining combined lifestyle behaviors rather than isolated factors. Moreover, compensatory behaviors—such as higher energy intake after exercise or reduced activity during dietary restriction—illustrate their interdependence [7]. Joint analyses of PA and diet have linked these behaviors to body composition, cardiometabolic outcomes, sleep, cognition, and psychosocial health [9,10,11,12,13].

Injury research, however, has emphasized PA-related determinants such as training volume, intensity, and sport type [14], while nutritional aspects have received far less attention. Yet, adequate nutrition is vital for adolescents and young adults facing high training demands and rapid growth [15]. Reductionist approaches focusing on isolated factors have limited predictive value [16]. Although clustering studies have described distinct combinations of diet, PA, and sedentary behaviors [17,18], they typically address general health outcomes rather than musculoskeletal injury, leaving an important gap.

Evidence is limited on how integrated lifestyle patterns—joint profiles of PA and dietary behaviors—relate to injury risk. Prior work has focused on physiological and biomechanical determinants [19], while little is known about how lifestyle factors interact to increase or reduce vulnerability [20]. For example, inadequate energy or protein intake combined with high PA loads may create unfavorable conditions for injury. Addressing this gap could provide new insight into the behavioral drivers of injury and inform more comprehensive prevention strategies. Unlike prior research focusing on isolated physiological or biomechanical predictors, this study integrates physical activity and dietary intake patterns to identify lifestyle clusters associated with injury occurrence, offering a broader behavioral perspective relevant to injury prevention.

Analytical approaches based on person-centered frameworks, including latent class analysis (LCA) and latent profile analysis (LPA), allow identification of lifestyle patterns that combine PA and diet [21,22]. Unlike variable-centered approaches, these techniques account for integrated behavioral constellations rather than isolated practices [23,24]. Applying them to musculoskeletal injuries may clarify how lifestyle profiles influence risk and support tailored prevention strategies. This approach is especially relevant in physically active young adults, who are at elevated risk yet remain understudied in this context.

Accordingly, the aim of this study was to (1) identify lifestyle profiles integrating PA and dietary intake (DI) in young adults, (2) assess whether lower limb injury prevalence differs across these profiles, and (3) evaluate their predictive value for lower limb injury risk using logistic regression adjusted for sex.

We hypothesized that distinct clusters of physical activity and dietary behaviors would emerge and that less favorable behavioral profiles would be associated with a higher prevalence of lower-limb injuries.

## 2. Materials and Methods

### 2.1. Study Design

This study employed a cross-sectional design and recruited a convenience sample of university students. Recruitment procedures and broader methodological details have been reported previously [25], while the present work represents a secondary analysis of that dataset [26]. This analysis focused specifically on the relationship between combined profiles of dietary quality and physical activity and their association with injury occurrence, applying latent profile modeling and regression-based methods.

Dietary information (frequency of healthy and unhealthy food consumption) and PA indicators (walking, moderate, and vigorous activity) were used to construct multidimensional lifestyle profiles. Injury history was self-reported and examined both as a binary outcome (injury: yes/no) and as a count variable (number of injuries) across profiles.

This design provided an integrated framework to evaluate behavioral and nutritional correlates of injury risk in physically active young adults.

### 2.2. Ethics

Ethical clearance was obtained from the Senate Research Ethics Committee of Wroclaw University of Health and Sport Sciences (reference number 13/2022). Before taking part in the research, each participant was fully informed about the study procedures and provided digital consent.

### 2.3. Sample Size

Sample size estimation followed recommended standards for exploratory multivariate analyses. A target of at least 240 individuals was established to provide sufficient statistical power for identifying clusters derived from 16 dietary and 4 physical activity indicators, as well as for conducting sex-specific logistic regression models. This target follows recommendations of at least 30 cases per expected profile and 10–20 cases per predictor for stable odds ratio estimates [27,28,29].

### 2.4. Participants

**Figure 1:**
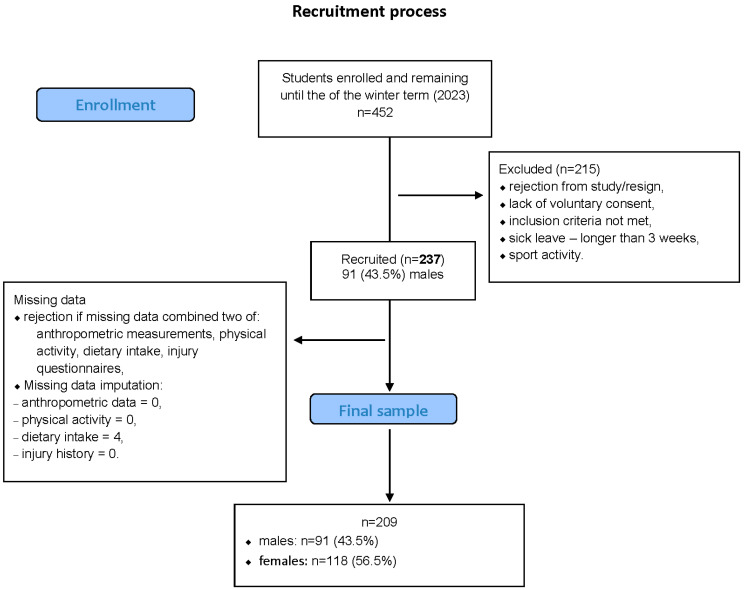
Flowchart of the recruitment process.

In total, 237 first-year students (44% men) enrolled in physical education and physiotherapy courses at Wroclaw University of Health and Sport Sciences took part in the study during the 2023 academic term. The sex distribution reflected that of the overall student population in these fields. Consistent with previous data [25], most participants reported high PA levels, with ≥3000 MET-min/week in 81% of men and 76% of women. The sampling process and final analytical sample are shown in Figure 1.

Participants were eligible if they (1) were enrolled in on-campus academic programs, (2) were under 22 years of age, and (3) provided informed consent. Additional criteria included residence in a city with >100,000 inhabitants (as a proxy for socioeconomic status), at least one parent with higher education, and a body mass index (BMI) classified as normal according to World Health Organization criteria.

Students were excluded if they engaged in university-level athletic programs, had taken medical leave longer than three weeks, or reported any ongoing illness or injury during data collection. Among the 237 initially eligible participants, 28 were removed because of incomplete data in several domains. Small deficiencies in dietary responses (*n* = 4) were subsequently corrected through the imputation method detailed below.

The final sample comprised 209 students (91 men, 118 women). Men averaged 183.6 cm in height, 79.2 kg in weight, and a BMI of 23.6 kg/m^2^, while women averaged 168.7 cm, 61.1 kg, and a BMI of 21.6 kg/m^2^. All values were within the normal weight range.

### 2.5. Data Collection

The data were obtained as part of the Family Lifestyle Patterns project (FAST-PAT23), focusing on physical activity, dietary habits, health-related attitudes, and socioeconomic background. Participants completed structured questionnaires through Google Forms directly following a scheduled Human Anatomy class. To maintain procedural uniformity, the same researcher conducted all stages of recruitment, questionnaire administration, and data entry. Anthropometric assessments were performed in person across four consecutive weeks in March 2023.

### 2.6. Anthropometric and Body Composition Measurements

Anthropometric measurements were conducted in the Biokinetics Research Laboratory at Wroclaw University of Health and Sport Sciences, certified according to Consort 2010 standard [30]. Body height was measured twice with a GPM anthropometer (accuracy 0.1 cm), and the average value was used for analysis. Body mass and composition were determined using the InBody230 bioelectrical impedance device (InBody Co., Ltd. (formerly Biospace Co., Ltd.), Seoul, Republic of Korea). BMI was calculated as weight (kg)/height^2^ (m^2^).

### 2.7. Questionnaire Measurements

#### 2.7.1. Physical Activity Questionnaire

PA was measured using the Polish adaptation of the International Physical Activity Questionnaire–Long Form (IPAQ-LF) [31], administered electronically via Google Forms. The questionnaire consists of 11 items covering domains such as work or study, transport, domestic and gardening activities, and leisure-time exercise, along with a separate question on sedentary time. Reported activities were expressed in MET-minutes per week to derive total physical activity, specific domain values, and time spent sitting. In this study, PA data were used only to verify comparable activity levels across participants, ensuring sample homogeneity and minimizing variability from training load.

#### 2.7.2. Dietary Intake Questionnaire

Dietary habits during the preceding 12 months were evaluated with the self-administered Questionnaire of Eating Behaviors (QEB) [32], a validated food frequency instrument demonstrating satisfactory reliability (Fleiss’ κ = 0.64–0.84). From the initial 21 items, the standard 16-item core version recommended for population studies was utilized. [33]. The QEB short form includes 16 food categories representing key indicators of healthy and unhealthy dietary habits. These items were used to identify overall eating patterns rather than quantify specific nutrients. Responses were provided on a six-point frequency scale ranging from “never” to “several times per day” and subsequently transformed into approximate daily intake equivalents. For this study, four components—fruits, vegetables, fast food, and sugar-sweetened beverages—were selected to construct a multidimensional dietary quality indicator (MDQI) and analyze dietary patterns in relation to injury occurrence.

#### 2.7.3. Recording of Musculoskeletal Injuries

Injuries were defined as self-reported musculoskeletal injuries sustained during physical activity, exercise, or sport within the previous 12 months. Information on injuries was gathered through the Injury History Questionnaire (IHQ), a tool previously validated and demonstrating strong measurement reliability. (Cronbach’s α = 0.836) [34]. The tool records injuries within the past 12 months by body region; for this study, only lower limb injuries were analyzed. Participants indicated whether they had experienced any musculoskeletal injury during physical activity in the past year and identified the affected body region. The majority of reported cases involved the lower limbs (ankle, knee, or thigh). Injury occurrence was dominated by lower-limb injuries (approximately 70% of all reported cases), whereas upper-limb and trunk injuries were less frequent. Upper-limb injuries were infrequent and thus not analyzed separately.

Injuries were coded by body region (head–neck–trunk, upper limb, lower limb) and by diagnosis (fracture, joint sprain, muscle/tendon strain, abrasion/skin wound, other). A detailed breakdown of diagnoses is provided in Supplementary Table S2, and the cross-tabulation of body region by diagnosis in Supplementary Table S3. Because multiple injuries per participant were possible, counts reflect injury instances, and percentages in Supplementary Table S2 may exceed 100% when referenced to injured participants. Injury counts and percentages in Supplementary Tables S2 and S3 refer to injury episodes (multiple episodes per participant were possible).

Data were self-reported via Google Forms, and completeness was checked against physical measurements. No missing data were observed.

### 2.8. Handling and Imputation of Missing Data

There were no missing observations for anthropometric, body composition, or physical activity variables. However, four participants provided incomplete dietary data in the QEB questionnaire. Because PCA and MDQI estimation rely on full datasets, these gaps were handled through imputation. The missingness pattern met the criteria for missing completely at random (MCAR) [35,36], suggesting the absence of systematic bias. Data imputation was carried out in R (RStudio v.2024.11.0) using the “mice” package (version 3.14.0, accessed 15 November 2024).

### 2.9. Statistics

Data from the IPAQ and QEB questionnaires were normalized using the Yeo–Johnson power transformation to achieve near-normal distributions [37]. Subsequently, all variables were standardized (mean = 0, SD = 1) to ensure scale comparability and to allow joint analysis of male and female participants. The Shapiro–Wilk test confirmed the normality of transformed variables, enabling the application of parametric descriptive statistics (means, 95% confidence intervals, and standard deviations). Descriptive statistics were computed for all PA and DI variables. Means and SDs were reported for continuous variables, and frequencies with percentages for categorical ones. Sex differences were tested with Student’s *t*-tests for independent samples. After normalization, male and female data were merged to increase analytical power, as small subgroup sizes would otherwise limit reliable inference.

Latent profile analysis (LPA) was applied to PA and DI variables to identify behavioral patterns. Latent profile analysis was performed using standardized PA and DI indicators to identify subgroups with similar behavioral patterns. This data-driven approach follows methods used in previous studies examining lifestyle clustering among youth and adults. Models with increasing class numbers were estimated, and selection was guided by Bayesian Information Criterion (BIC), entropy, and class interpretability. Posterior probabilities were examined to assess classification accuracy. For the optimal solution, standardized means (z-scores) were calculated for each component, indicating deviations from the sample mean. The five largest deviations per profile were used to describe its defining features. Multivariate differences between profiles were tested with PERMANOVA (999 permutations, Euclidean distance), with Holm-adjusted *p*-values and R^2^ reported. Participants were assigned to the class with the highest posterior probability.

Differences in injury occurrence across profiles were tested with chi-square analyses; Fisher’s exact test was applied when expected counts were <5. Multivariable logistic regression was then used to assess whether profile membership predicted injury, adjusting for sex, age, and BMI. In the case of the subgroup that does not meet the conventional “8–10 cases per predictor” rule (often cited in logistic regression analyses), we treated this guideline as a heuristic rather than a strict requirement. Several methodological studies have noted that the EPV ≥ 8 criterion is not a universally valid threshold, particularly in small-sample contexts, and that factors such as bias, separation, and data structure may play an equal or greater role than the simple ratio of events to predictors As a robustness check, we performed a sensitivity analysis using Firth’s bias-reduced logistic regression, which is designed to mitigate small-sample bias and improve parameter stability in sparse data. For parsimony and stability in this sensitivity analysis, the PA–DI profile was additionally parameterized as an ordinal trend (1–4) alongside the categorical coding used in the main models. The direction and relative magnitude of coefficients were comparable to standard maximum-likelihood estimates, with wider profile-likelihood confidence intervals as expected in small samples. An appropriate note regarding this issue has been added to the Limitations section. Associations were expressed as odds ratios (OR) with 95% confidence intervals (CI). Statistical significance was set at *p* < 0.05.

Handling of PA as a covariate. Physical activity indicators (IPAQ-LF) were incorporated directly into the latent class model as components of the integrated PA–DI profiles. Therefore, re-introducing PA as a separate covariate in logistic regression would be redundant and could induce multicollinearity and over-adjustment. Accordingly, regression models were adjusted for sex, age, and BMI, while PA effects were captured at the profile level.

All analyses used a significance threshold of α = 0.05. Calculations were performed in Statistica 13.5 (StatSoft, Cracow, Poland) and RStudio 2025.05.0+496 (Posit Software, Boston, MA, USA), with latent class modeling conducted via the lcmm package (v. 2.2.1).

Language editing: The English language of the manuscript was revised using an AI-based tool (ChatGPT, GPT-5, OpenAI, San Francisco, CA, USA) to improve clarity and grammar. The authors reviewed and edited the content as needed, and take full responsibility for the final version of the manuscript.

## 3. Results

### 3.1. Basic Descriptive Characteristics of the Low- and High-Distress Groups

Men were significantly taller, heavier, and had lower body fat than women (all *p* < 0.001). In IPAQ domains, differences appeared for moderate activity (*p* = 0.010) and sitting time (*p* = 0.006), whereas vigorous and walking activity did not differ. For dietary variables (QEB), only curd cheese intake was higher in men (*p* = 0.016); no other food groups varied significantly between sexes (Table 1).

Among 209 participants, 123 (58.9%) reported at least one injury, totaling 363 injury episodes. Most injuries involved the lower limbs (227; 62.5%), followed by the upper limbs (107; 29.5%) and the neck/head/trunk (29; 8.0%). By type, muscle strains (130; 35.8%) and joint sprains (107; 29.5%) were most frequent, with skin abrasions (95; 26.2%) and fractures (31; 8.5%) less common. A detailed breakdown by diagnosis and by body region is provided in Supplementary Tables S2 and S3.

Because dietary and PA profiles were broadly comparable between sexes, data were normalized, standardized, and combined into a single analytic group to ensure sufficient power for latent profile analysis.

### 3.2. Identifiying Latent Profiles of the Dietary and Physical Activity Behaviors

Latent profile modeling of PA and DI components tested solutions with two to six classes. Model fit indices (log-likelihood, AIC, BIC, entropy) are presented in Table 2. The four-class model was selected as optimal, showing the lowest BIC (11,332.12), high entropy (0.931), and good interpretability. Average posterior probabilities ranged between 0.84 and 0.92 across profiles, indicating good classification accuracy and low uncertainty (entropy = 0.83).

**Figure 2:**
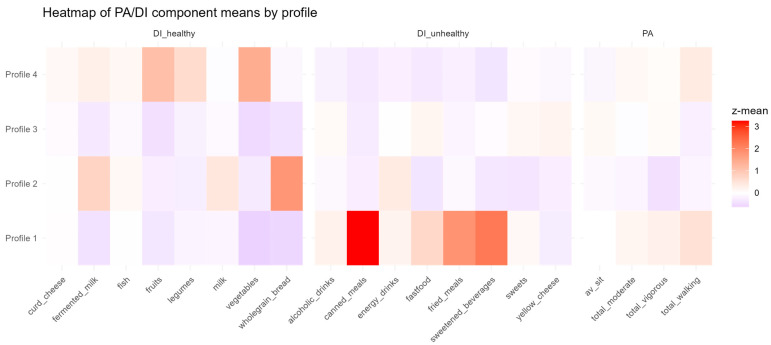
Heatmap of mean standardized scores (z-scores) for physical activity (PA) and dietary intake (DI) components across four latent profiles. Warmer colors (red) indicate higher relative values and cooler colors (blue) indicate lower values. Profiles differ in their combinations of healthy DI, unhealthy DI, and PA, illustrating distinct lifestyle behavior patterns.

The heatmap (Figure 2) displays mean standardized PA and DI values across the four profiles. Clear contrasts emerged, with some profiles marked by greater sedentary behavior and unhealthy food intake, and others by healthier diets and higher PA. This visualization underscores the distinct configurations defining each profile and supports their interpretability.

In addition, Table 3 presents the five most distinctive components for each profile, identified as those with the largest absolute deviations from the overall sample mean (z-score).

Profile 1 (*n* = 17, 8.1%) showed a generally healthy pattern, with higher vegetable (+1.05 SD) and fruit (+0.82 SD) intake, more vigorous PA (+0.75 SD), and lower sweets (−0.64 SD) and energy drink consumption (−0.55 SD).

Profile 2 (*n* = 34, 16.3%) was less favorable, marked by greater alcohol (+0.98 SD) and fast-food intake (+0.77 SD), and lower consumption of legumes (–0.61 SD), vegetables (−0.59 SD), and fruits (−0.52 SD).

Profile 3 (*n* = 104, 49.8%) represented the least healthy cluster, with higher sedentary time (+1.12 SD), sweetened beverages (+0.83 SD), and fried meals (+0.71 SD), combined with lower milk (−0.65 SD) and walking (−0.58 SD).

Profile 4 (*n* = 54, 25.8%) was the most health-oriented, showing higher intake of vegetables (+1.20 SD), legumes (+0.92 SD), and fermented milk (+0.81 SD), along with lower fast-food (−0.73 SD) and sugary drink intake (−0.62 SD).

PERMANOVA showed significant overall differences in PA–DI structures across the four profiles (F(3,205) = 18.24, R^2^ = 0.211, *p* = 0.001). Pairwise tests (Holm-adjusted) indicated that all profiles differed significantly (*p* < 0.01). The largest contrasts were between Profile 1 and Profiles 2 (R^2^ = 0.291) and 4 (R^2^ = 0.238), while smaller but significant differences were observed between Profiles 2 vs. 3 (R^2^ = 0.093) and 3 vs. 4 (R^2^ = 0.112).

### 3.3. Injuries Prevalence Across the Profiles in Best Fitted Model

In total, 119 participants (56.9%) reported injuries. Prevalence was 52.9% in Profile 1, 44.1% in Profile 2, 60.6% in Profile 3, and 66.7% in Profile 4 (Table 4).

The overall chi-square test showed no significant differences between profiles (χ^2^ (3) = 4.78, *p* = 0.188). Pairwise analysis indicated higher injury prevalence in Profile 4 vs. Profile 2 (66.7% vs. 44.1%; χ^2^ (1) = 5.08, *p* = 0.024; Fisher’s exact *p* = 0.027), with no other profile differences (*p* > 0.15).

### 3.4. Sex Effect in Injury Occurence in Relation to Profile of Dietary and Physical Activity Behaviors

**Figure 3:**
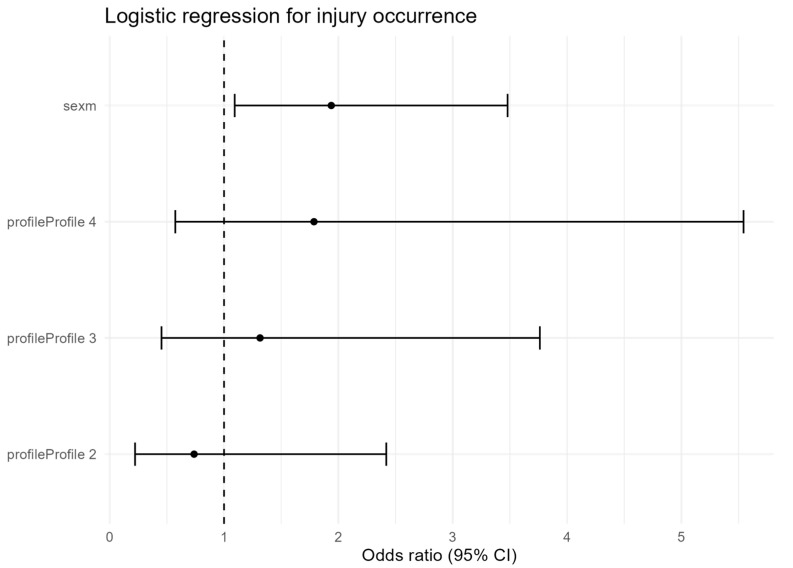
Odds ratios (OR) with 95% confidence intervals for injury occurrence predicted by sex and PA–DI profile membership. Profile 1 (reference group) and female sex were used as reference categories. Male sex was associated with significantly higher injury risk, whereas differences between profiles did not reach statistical significance.

Logistic regression controlling for PA–DI profile showed that men had nearly twice the odds of injury compared with women (OR = 1.94, 95% CI: 1.09–3.48, *p* = 0.025) (Table 5, Figure 3). Profile membership was not a significant predictor; however, odds were directionally higher in Profiles 3 (OR = 1.31, 95% CI: 0.45–3.76) and 4 (OR = 1.79, 95% CI: 0.57–5.54) compared with Profile 1. Estimates for smaller profiles (e.g., Profile 1, *n* = 17) should be interpreted with caution due to limited precision.

Note that PA indicators are part of the latent profiles; thus, models were adjusted for sex, age, and BMI only to avoid redundancy and multicollinearity. As a sensitivity check, Firth’s bias-reduced logistic regression (with the profile also parameterized ordinally, 1–4) yielded comparable coefficient directions and inferences to the ML estimates; profile-likelihood confidence intervals were wider, and conclusions remained unchanged.

When the interaction term profile × sex was included, no significant effects emerged, indicating that the influence of sex on injury risk was consistent across all lifestyle profiles (Table 6).

These associations should be interpreted cautiously given the cross-sectional design and exploratory nature of the analysis. The same pattern held for the profile × sex interaction model under Firth’s correction.

## 4. Discussion

This study examined how integrated lifestyle behaviors—combinations of physical activity (PA) and dietary intake (DI)—relate to injury risk in physically active young adults. Latent profile analysis identified four distinct lifestyle profiles that differed significantly in multivariate structure (PERMANOVA). Two profiles reflected less healthy patterns, while two were more favorable. Unexpectedly, injury prevalence did not follow a simple gradient: the healthiest profile, marked by high vegetable, legume, and fermented milk intake with low fast-food and sugary drinks, showed the highest injury rate, whereas one of the less favorable profiles had the lowest. Logistic regression further revealed that sex, not profile membership, was the strongest predictor, with men nearly twice as likely as women to report injuries. These findings underscore both the value and complexity of integrating PA and DI patterns to understand injury vulnerability.

The paradoxical finding that the healthiest profile showed higher injury prevalence requires careful interpretation. While no direct link between healthy eating and greater injury risk is evident, individuals with health-oriented lifestyles may also engage in more vigorous training, structured exercise, or competitive sports. Such behaviors, though beneficial for fitness, increase musculoskeletal injury risk through higher exposure [16,38,39]. The paradoxical finding that the healthiest profile showed a higher prevalence of injury may reflect greater exposure to training and competitive physical activity. Students with high health orientation and performance motivation may also display perfectionistic or overcontrolled behaviors, increasing susceptibility to overuse or stress-related injuries despite generally favorable health behaviors. Consistent with prior evidence, training volume, intensity, and load management remain among the strongest predictors of sports injuries [14]. Thus, the elevated risk in Profile 4 likely reflects the dual role of PA: essential for health, yet a source of mechanical and physiological stress that can predispose to injury.

Conversely, Profile 2—with less favorable dietary habits—showed the lowest injury prevalence, suggesting that lower engagement in vigorous PA may act protectively in some subgroups. This aligns with the multifactorial injury model by Bittencourt et al. [16], which highlights interactions between intrinsic and extrinsic factors, and supports the utility of behavioral clustering over single-variable analyses. Indeed, Mello et al. (2021) [40] identified 55 global PA–diet–sedentary clusters, while Matias et al. (2018) [41] found three distinct patterns among Brazilian adolescents, with a quarter engaging in multiple risk behaviors. Similarly, Russell et al. (2016) [42] showed that risk-taking behavior clusters in Canadian youth were linked to injury, especially in unsupervised environments. Prospective data from Chinese students further confirm that greater weekly PA exposure significantly raises injury incidence [43], emphasizing that activity load can outweigh potential dietary protection.

Sex differences emerged as one of the strongest findings: men were nearly twice as likely as women to report injuries, independent of profile membership. Prior research also shows higher overall injury rates in males, including greater risk of hip/groin, hamstring, upper-limb, and posterior thigh overuse injuries [44,45,46]. Explanations include biological factors (muscle mass, hormonal milieu, joint morphology) [2], behavioral tendencies (greater risk-taking, vigorous or contact sport participation) [3,4], and cultural influences that reinforce competitiveness among men.

These results support prior evidence that male sex is a consistent predictor of musculoskeletal injury in active populations [47,48]. Still, certain injuries, notably anterior cruciate ligament (ACL) tears, are reported more often in women due to biomechanical and neuromuscular factors [19]. Anatomical differences in pelvic and lower-limb morphology, combined with neuromuscular and hormonal influences, contribute to sex-specific injury patterns [49]. Thus, injury risk is complex and context-dependent: while women may be predisposed to ACL injury, men show higher overall injury rates. Our findings reinforce the need for sex-specific prevention strategies, with male sex emerging as the dominant risk factor in this university sample.

Although diet is critical for musculoskeletal integrity, this study found no independent link between dietary quality and injury once sex and activity profile were considered. Sufficient consumption of protein, calcium, vitamin D, and total energy promotes bone and muscle regeneration, potentially lowering the likelihood of injury [15], but such effects may be subtle or obscured in cross-sectional analyses, particularly in homogeneous samples with high PA levels and normal BMI.

Recent evidence shows that nutritional deficiencies can interact with training demands to raise injury risk. A systematic review and meta-analysis reported that female runners with low energy and fat intake were more prone to musculoskeletal injuries, while insufficient dietary fiber increased risk in both sexes [20]. Our results are consistent with this view, suggesting that the limited dietary indicators assessed here (fruits, vegetables, fast food, sugary drinks) may not capture the nutrients most relevant for injury prevention. Future work should therefore incorporate detailed measures of energy intake, macronutrient distribution, and micronutrient adequacy.

Beyond physiological mechanisms, lifestyle interactions also warrant attention. Khoshro and Farhangi (2024) [50] observed that commitment to healthy dietary patterns was unexpectedly associated with stronger tendencies toward exercise dependence and body image preoccupation among physically active young adults. This indicates that beneficial eating behaviors may coexist with psychological or behavioral traits that indirectly heighten injury susceptibility. Additional behavioral determinants—such as older age, smoking, and low self-rated PA—have been identified in military trainees [51]. More broadly, McIntosh (2004) [52] emphasized the value of integrated frameworks that combine medical, behavioral, physiological, and biomechanical perspectives to explain injury causation. Together with nutritional evidence, these insights highlight the need for multifactorial, holistic prevention strategies that address both biological needs and complex lifestyle constellations.

The identification of distinct lifestyle profiles aligns with prior research using latent class or profile analysis to examine health behaviors in adolescents and young adults [17,18,21,22]. These studies show that PA and DI commonly cluster in synergistic or opposing ways, producing patterns with differing health implications. For instance, Ottevaere et al. (2011) [17] reported adolescent clusters combining low PA with unhealthy diets, as well as high PA with healthier eating. Similarly, Miranda et al. (2020) [53] linked integrated PA–DI profiles to cardiometabolic risk markers, underscoring interactions between body composition, inflammation, and lifestyle.

Among university students, Bennasar-Veny et al. (2020) [54] identified lifestyle clusters ranging from “healthy” to “unhealthy high risk,” illustrating the heterogeneity of young adults’ behaviors. Similar clustering has been observed in occupational settings, such as Uruguayan workers, where distinct PA–diet patterns showed different health implications [55]. Systematic reviews further confirm that PA, diet, and sedentary behavior cluster consistently across populations, shaping both physical and mental health outcomes [56].

Most clustering studies have focused on outcomes such as obesity, cardiometabolic risk, mental health, or academic performance [9,10,11,12,13,21,22], with few addressing musculoskeletal injuries. A key strength of this study is extending behavioral profiling into this domain, offering initial evidence that injury risk may also differ across integrated lifestyle clusters. At the same time, the stronger effect of sex compared with profile membership underscores that clustering explains only part of the variability, and biological as well as behavioral sex differences must be considered in parallel.

These findings carry several practical implications. First, injury prevention should not assume that healthy diet plus high PA lowers risk; high activity volumes, even with favorable nutrition, may increase exposure and injury likelihood. Training programs should therefore emphasize balanced loads, recovery, and gradual progression, particularly for those engaged in high-intensity routines. Second, interventions should target integrated lifestyle profiles rather than single behaviors, with brief PA–DI screening tools offering a way to identify higher-risk students. Finally, sex-specific strategies are essential: men may benefit from tailored programs emphasizing load management and safe training practices, while in women, ensuring nutritional adequacy (e.g., preventing RED-S) remains critical despite lower overall injury prevalence. Because of the cross-sectional design, the findings indicate associations rather than causal relationships. The study should therefore be viewed as exploratory, generating hypotheses for future longitudinal research.

Several methodological limitations should be acknowledged in this study. The cross-sectional design precludes causal inference; it is possible that individuals who were previously injured subsequently improved their dietary or activity habits, leading to a reversed association between healthier profiles and higher injury prevalence. Longitudinal studies are needed to establish whether lifestyle patterns precede injuries or result from them. The study population consisted of physical education and physiotherapy students, who represent a highly active and health-oriented academic subgroup. This specificity should be considered when interpreting the findings, as results may not generalize to less active or non-sport university populations The sample consisted mainly of normal-weight students, which may limit generalizability to underweight or overweight populations. In addition, the use of the self-reported International Physical Activity Questionnaire–Long Form (IPAQ-LF) may have introduced measurement bias compared with objective assessments such as accelerometry. The IPAQ-LF provides global estimates of activity volume but does not capture sport-specific training variables such as session intensity, duration, or competitive load, which may influence injury risk. Because PA variables were part of the profile structure, they were not reintroduced as covariates in regression models; future studies using alternative modeling strategies could explicitly control for activity volume. Because the dietary tool assessed frequency rather than quantity, energy and protein intake could not be estimated. Future studies should include detailed nutritional assessments to capture energy availability and macronutrient balance. Although both IPAQ-LF and QEB are validated instruments, self-report data are inherently prone to recall bias and may underestimate or overestimate actual behavior and injury occurrence. A key limitation is the small sample size in Profile 1 (*n* = 17), which falls short of the classical heuristic EPV ≥ 10 threshold. This may lead to inflated estimates, wide confidence intervals, and sensitivity to model specification. Although we used penalized regression as a sensitivity check (or proponujemy to w przyszłych badaniach), the results for this subgroup should be interpreted cautiously. More detailed measures, including energy availability and micronutrient intake, would improve accuracy. Third, the sample consisted of physically active university students with normal BMI and relatively homogeneous backgrounds, limiting generalizability to adolescents, elite athletes, or sedentary populations. Although adequate for clustering, the sample size reduced power for regression models. Finally, profiles were based on a limited set of PA and DI variables, which improved interpretability but may have oversimplified behavior patterns. Future research should integrate additional lifestyle domains such as sleep, stress, and substance use. Additionally, future studies should incorporate detailed nutrient-level assessments, such as protein, calcium, and vitamin D intake, which are relevant to musculoskeletal health.

## 5. Conclusions

In summary, the findings of this study indicate that physically active young adults can be grouped into distinct PA–DI profiles with clear behavioral differences. Although injury prevalence varied across profiles, male sex was the most consistent predictor of injury. The paradoxical finding that the healthiest profile had the highest injury rate highlights the complexity of lifestyle–injury interactions and underscores the need for integrated, sex-sensitive prevention strategies.

Future work should: (1) employ longitudinal designs with prospective injury surveillance to establish causality; (2) include detailed dietary and physiological measures to clarify nutrition–injury links; and (3) conduct sport-specific and sex-stratified analyses to capture nuanced risk patterns. Embedding lifestyle-based risk assessment into university and sports programs may help reduce injuries while promoting healthier long-term behaviors.

## Figures and Tables

**Table 1:** Baseline characteristics of participants by sex (data transformed and standardized before analysis). T- and *p*-values were obtained from independent-sample Student’s *t*-tests, with statistically significant differences highlighted in bold.

		Males (*n* = 91)				Females (*n* = 118)					
Variable		Mean	95% CI		SD	Mean	95% CI		SD	t	*p*
			Lower	Upper			Lower	Upper			
age		20.5	20.2	20.7	1.0	20.3	20.2	20.5	0.8	0.79	0.429
	Body height [cm]	183.6	182.2	185.1	7.1	168.7	167.8	169.7	5.3	**17.41**	***p* < 0.001**
Anthropometry	Body weight [kg]	79.2	77.1	81.3	10.0	61.1	59.6	62.7	8.4	**14.17**	***p* < 0.001**
	BMI [kg/m^2^]	23.6	23.1	24.1	2.4	21.6	21.1	22.1	2.7	**−12.75**	***p* < 0.001**
	Fat mass [%]	15.3	14.4	16.2	4.2	23.9	23.0	24.9	5.3	**5.45**	***p* < 0.001**
	Walking intensity	0.0	−0.2	0.2	1.0	0.0	−0.2	0.2	1.0	0.02	0.986
	Moderate intensity	0.2	0.0	0.4	1.0	−0.2	−0.3	0.0	1.0	**2.59**	**0.010**
IPAQ	Vigorous intensity	0.1	−0.2	0.3	1.0	0.0	−0.2	0.1	1.0	0.66	0.510
	Sitting average	−0.2	−0.4	0.0	0.9	0.2	0.0	0.4	1.0	**−2.79**	**0.006**
	Wholebread	−0.1	−0.3	0.1	0.8	0.1	−0.1	0.3	1.1	−1.49	0.137
	Milk	0.0	−0.2	0.2	1.1	−0.1	−0.2	0.1	0.9	0.58	0.566
	Fermented milk	0.0	−0.2	0.2	1.0	0.0	−0.2	0.2	1.0	−0.20	0.839
	Curd cheese	0.2	−0.1	0.4	1.1	−0.1	−0.3	0.0	0.8	**2.43**	**0.016**
	Fish	0.1	−0.2	0.3	1.2	−0.1	−0.2	0.1	0.8	1.05	0.297
QEB	Legumes	0.0	−0.2	0.3	1.2	0.0	−0.2	0.1	0.9	0.25	0.806
	Fruits	−0.1	−0.3	0.1	0.9	0.1	−0.1	0.3	1.1	−1.71	0.089
	Vegetables	−0.1	−0.3	0.1	1.0	0.0	−0.1	0.2	1.0	−0.86	0.393
	Fastfood	0.0	−0.2	0.2	1.0	0.0	−0.2	0.2	1.0	−0.17	0.865
	Fried meals	0.1	−0.1	0.2	0.9	0.0	−0.2	0.2	1.1	0.17	0.864
	Yellow cheese	−0.1	−0.3	0.1	0.9	0.1	−0.1	0.3	1.0	−1.40	0.163
	Sweets	−0.1	−0.3	0.1	0.8	0.1	−0.1	0.3	1.1	−1.56	0.119
	Canned meals	0.0	−0.2	0.2	1.0	0.1	−0.1	0.2	1.0	−0.59	0.559
	Sweetened beverages	0.0	−0.2	0.1	0.9	0.0	−0.2	0.2	1.1	−0.63	0.532
	Energetic drinks	−0.1	−0.3	0.1	0.8	0.0	−0.2	0.2	1.1	−0.85	0.395
	Alcoholic drinks	−0.1	−0.2	0.1	0.8	0.0	−0.2	0.3	1.1	−0.78	0.438

Footnote. IPAQ—International Physical Activity Questionnaire, QEB—Questionnaire Eating Behavior, CI—Confidence Interval, SD—Standard Deviation. Significance codes: bold font—*p* < 0.05.

**Table 2:** Fit indices for latent class mixed models (2–6 class solutions).

Model	Number of Classes	BIC	Entropy
2-class	2	11,464.88	1.000
3-class	3	11,472.92	0.942
4-class	4	11,332.12	0.931
5-class	5	11,333.59	0.931
6-class	6	11,362.52	0.933

**Table 3:** Top five components distinguishing each latent profile of physical activity and dietary intake, expressed as mean standardized scores (z-scores).

Profile	Variable (Component)	z-Mean
1	Vegetables	+1.05
	Fruits	+0.82
	Vigorous PA	+0.75
	Sweets	−0.64
	Energy drinks	−0.55
2	Alcoholic drinks	+0.98
	Fast food	+0.77
	Legumes	−0.61
	Vegetables	−0.59
	Fruits	−0.52
3	Sitting time	+1.12
	Sweetened beverages	+0.83
	Fried meals	+0.71
	Milk	−0.65
	Walking	−0.58
4	Vegetables	+1.20
	Legumes	+0.92
	Fermented milk	+0.81
	Fast food	−0.73
	Sweetened beverages	−0.62

**Table 4:** Frequency and prevalence of self-reported injuries by profiles.

Profile	Injury	
	0	1
1	8 (47.1%)	9 (52.9%)
2	19 (55.9%)	15 (44.1%)
3	41 (39.4%)	63 (60.6%)
4	18 (33.3%)	36 (66.7%)

**Table 5:** Logistic regression models predicting injury occurrence by PA–DI profile, adjusted for sex, age, and BMI.

Term	Std. Error	Statistic	*p*-Value	OR	−95% CI	95% CI
profile: Profile 2	0.604	−0.503	0.615	0.738	0.222	2.42
profile: Profile 3	0.533	0.514	0.607	1.31	0.453	3.76
profile: Profile 4	0.572	1.01	0.311	1.79	0.574	5.54
sex: m	0.295	2.24	0.0248	1.94	1.09	3.48

**Table 6:** Logistic regression model with profile × sex interaction predicting injury occurrence. Odds ratios (OR) with 95% confidence intervals (CI) and *p*-values are reported. Female sex and Profile 1 served as the reference categories.

Term	Std. Error	Statistic	*p*-Value	OR	−95% CI	95% CI
Profile 2 (Female vs. Profile 1)	0.763	−0.579	0.563	0.643	0.139	2.920
Profile 3 (Female vs. Profile 1)	0.689	0.108	0.914	1.080	0.271	4.290
Profile 4 (Female vs. Profile 1)	0.732	0.628	0.530	1.580	0.368	6.870
Sex: Male (vs. Female, Profile 1)	0.992	0.290	0.772	1.330	0.189	10.100
Interaction: Profile 2 × Male	1.240	0.272	0.786	1.400	0.119	16.100
Interaction: Profile 3 × Male	1.070	0.452	0.651	1.620	0.186	13.400
Interaction: Profile 4 × Male	1.160	0.254	0.800	1.340	0.132	13.200

## Data Availability

The raw data supporting the conclusions of this article will be made available by the author on request.

## Supplementary Materials

The following supporting information can be downloaded at: https://www.mdpi.com/article/10.3390/nu17203196/s1, Table S1.A: Whole group, without sex separation; Table S1.B: Male group; Table S1.C: Female group; Table S2: Breakdown of actual injury diagnoses by type (n, % of injured participants; multiple injuries per participant possible); Table S3: Injury type by body region (counts of injury instances; multiple injuries per participant possible; row/column totals shown).
