# Supplementary material for: Integrated Behavioral Profiles of Physical Activity and Dietary Intake in Young Adults and Their Associations with Lower Limb Injury Occurrence
Source: Nutrients. 2025 Oct 11;17(20):3196. doi: 10.3390/nu17203196 (PMC12567443; doi:10.3390/nu17203196)
Supplement: Supplementary file 1 [file nutrients-17-03196-s001.zip › Supplementary ST1.pdf]

Supplementary file

Table S1.A. Whole group, without sex separation. Descriptive statistics for physical activity (IPAQ) and dietary intake (QEB) variables (non-transformed data), including means, 95% confidence intervals, standard deviations (SD), medians, interquartile ranges (IQRs) and skewness.

Variable	Mean	Lower CI (95%)	Upper CI (95%)	Median	IQR	SD	Skewness
Wholegrain.bread	0,30	0,25	0,34	0,10	0,30	0,33	1,64
Milk	0,42	0,37	0,47	0,40	0,70	0,37	1,23
Fermented.milk	0,39	0,35	0,43	0,40	0,30	0,29	1,28
Curd.cheese	0,19	0,17	0,22	0,10	0,30	0,17	1,29
Fish	0,10	0,09	0,11	0,10	0,00	0,09	4,10
Legumes	0,13	0,11	0,15	0,10	0,00	0,16	4,32
Fruits	0,56	0,50	0,61	0,40	0,40	0,42	1,17
Vegetables	0,69	0,62	0,75	0,40	0,40	0,47	0,81
Fastfood	0,14	0,12	0,16	0,10	0,00	0,12	3,08
Fried.meals	0,37	0,33	0,41	0,40	0,30	0,29	1,80
Yellow.cheese	0,39	0,35	0,43	0,40	0,30	0,32	1,47
Sweets	0,40	0,35	0,45	0,40	0,30	0,35	1,80
Canned.meals	0,10	0,07	0,13	0,00	0,10	0,22	2,75
Sweetened.beverages	0,19	0,15	0,22	0,10	0,00	0,26	2,41
Energy.drinks	0,14	0,11	0,17	0,10	0,10	0,20	2,89
Alcoholic.drinks	0,09	0,08	0,11	0,10	0,00	0,08	4,76
Walk	79,54	74,30	84,78	72,50	43,20	38,43	1,50
Moderate	83,65	78,22	89,08	77,30	50,80	39,82	0,62
Vigorous	81,93	74,65	89,22	78,00	68,40	53,44	0,33
Average sit	32,72	31,80	33,65	31,50	8,70	6,80	1,19

Table S1.B. Male group. Descriptive statistics for physical activity (IPAQ) and dietary intake (QEB) variables (non-transformed data), including means, 95% confidence intervals, standard deviations (SD), medians, interquartile ranges (IQRs) and skewness.

Variable	Mean	Lower CI (95%)	Upper CI (95%)	Median	IQR	SD	Skewness
Wholegrain.bread	0,26	0,20	0,31	0,10	0,30	0,28	1,63
Milk	0,44	0,35	0,52	0,40	0,30	0,41	1,42
Fermented.milk	0,38	0,32	0,45	0,40	0,30	0,30	1,74
Curd.cheese	0,23	0,19	0,27	0,10	0,30	0,20	1,23
Fish	0,11	0,09	0,13	0,10	0,00	0,10	4,33
Legumes	0,14	0,10	0,18	0,10	0,00	0,19	4,61
Fruits	0,50	0,42	0,58	0,40	0,40	0,38	1,28
Vegetables	0,65	0,56	0,75	0,40	0,40	0,46	0,93
Fastfood	0,14	0,12	0,17	0,10	0,00	0,12	2,89
Fried.meals	0,38	0,32	0,43	0,40	0,30	0,25	1,33
Yellow.cheese	0,36	0,30	0,42	0,40	0,30	0,29	1,49
Sweets	0,35	0,30	0,41	0,40	0,30	0,27	1,79
Canned.meals	0,09	0,05	0,13	0,00	0,10	0,21	2,99
Sweetened.beverages	0,18	0,13	0,23	0,10	0,00	0,22	1,84
Energy.drinks	0,13	0,10	0,16	0,10	0,10	0,16	2,19
Alcoholic.drinks	0,09	0,08	0,10	0,10	0,00	0,06	2,59
Walk	79,61	71,77	87,46	72,50	46,30	37,67	1,20
Moderate	91,63	83,10	100,15	85,00	59,20	40,94	0,58
Vigorous	84,62	73,43	95,81	81,90	66,30	53,72	0,30
Average sit	31,22	29,92	32,52	30,10	6,90	6,25	1,72

Table S1.C. Female group. Descriptive statistics for physical activity (IPAQ) and dietary intake (QEB) variables (non-transformed data), including means, 95% confidence intervals, standard deviations (SD), medians, interquartile ranges (IQRs) and skewness.

Variable	Mean	Lower CI (95%)	Upper CI (95%)	Median	IQR	SD	Skewness
Wholegrain.bread	0,33	0,26	0,40	0,10	0,30	0,37	1,52
Milk	0,40	0,34	0,47	0,40	0,70	0,34	0,92
Fermented.milk	0,39	0,34	0,44	0,40	0,30	0,27	0,83
Curd.cheese	0,17	0,14	0,19	0,10	0,30	0,14	0,90
Fish	0,09	0,08	0,11	0,10	0,00	0,07	2,65
Legumes	0,13	0,10	0,15	0,10	0,00	0,13	2,45
Fruits	0,60	0,52	0,68	0,40	0,40	0,45	1,06
Vegetables	0,71	0,62	0,80	0,40	0,40	0,48	0,73
Fastfood	0,14	0,12	0,16	0,10	0,00	0,13	3,24
Fried.meals	0,37	0,32	0,43	0,40	0,30	0,32	1,94
Yellow.cheese	0,42	0,36	0,48	0,40	0,30	0,33	1,44
Sweets	0,43	0,36	0,50	0,40	0,30	0,39	1,64
Canned.meals	0,11	0,07	0,15	0,00	0,10	0,22	2,63
Sweetened.beverages	0,20	0,15	0,25	0,10	0,00	0,28	2,54
Energy.drinks	0,15	0,11	0,19	0,10	0,10	0,23	2,92
Alcoholic.drinks	0,10	0,08	0,11	0,10	0,00	0,09	4,96
Walk	79,49	72,35	86,63	72,95	40,00	39,16	1,71
Moderate	77,50	70,58	84,42	73,90	42,60	37,98	0,65
Vigorous	79,86	70,13	89,59	77,50	72,00	53,35	0,36
Average sit	33,89	32,61	35,16	32,70	9,20	7,01	0,91
