# Supplementary material for: Integrated Behavioral Profiles of Physical Activity and Dietary Intake in Young Adults and Their Associations with Lower Limb Injury Occurrence
Source: Nutrients. 2025 Oct 11;17(20):3196. doi: 10.3390/nu17203196 (PMC12567443; doi:10.3390/nu17203196)
Supplement: Supplementary file 1 [file nutrients-17-03196-s001.zip › Supplementary_ST2.pdf]

Supplementary file – ST2

Table S2 Breakdown of actual injury diagnoses by type (n, % of injured participants; multiple injuries per participant possible).

Injury type	n	%
Fracture	31	25,2
Joint sprain	107	87
Muscle/tendon strain	130	105,7
Abrasion/skin wound	95	77,2
