# Supplementary material for: Integrated Behavioral Profiles of Physical Activity and Dietary Intake in Young Adults and Their Associations with Lower Limb Injury Occurrence
Source: Nutrients. 2025 Oct 11;17(20):3196. doi: 10.3390/nu17203196 (PMC12567443; doi:10.3390/nu17203196)
Supplement: Supplementary file 1 [file nutrients-17-03196-s001.zip › Supplementary_ST3.pdf]

Supplementary file – ST3

Table S3 Injury type by body region (counts of injury instances; multiple injuries per participant possible; row/column totals shown).

Body Region	Fracture	Joint sprain	Muscle/tendon strain	Abrasion/ skin wound	Total
Head–neck–trunk	7	8	1	5	21
Upper limb	6	15	13	14	48
Lower limb	14	20	12	22	68
Total	27	43	26	41	137
